# Liver and plasma lipid changes induced by cyclic fatty acid monomers from heated vegetable oil in the rat

**DOI:** 10.1002/fsn3.766

**Published:** 2018-10-16

**Authors:** Jean Mboma, Nadine Leblanc, Sereana Wan, René L. Jacobs, André Tchernof, Pascal Dubé, Paul Angers, Hélène Jacques

**Affiliations:** ^1^ School of Nutrition Laval University Quebec City Quebec Canada; ^2^ Institute of Nutrition and Functional Foods Laval University Quebec City Quebec Canada; ^3^ Department of Biochemistry University of Alberta Edmonton Alberta Canada; ^4^ Department of Agricultural, Food and Nutritional Science 4‐002 Li Ka Shing Centre for Health Research Innovations University of Alberta Edmonton Alberta Canada; ^5^ Quebec Heart and Lung Institute Quebec City Quebec Canada; ^6^ Department of Food Science Laval University Quebec City Quebec Canada

**Keywords:** cholesterol homeostasis, cyclic fatty acid monomers, fatty liver, male Wistar rats, phosphatidylcholine biosynthesis

## Abstract

Cyclic fatty acid monomers (CFAM) generated through domestic or industrial heating of vegetable oils may alter liver enzymes and induce hepatomegaly and steatosis, but the underlying mechanisms are not clearly understood. This study aimed to assess the effects of CFAM on liver and plasma lipids and to determine whether these effects are modulated by dietary lipids. Thirty‐six (36) male Wistar rats were fed either of the four isoenergetic diets consisting of canola oil or soybean oil with/without 500 mg/100 g CFAM of total fat for 28 days. Rats fed CFAM had higher liver total lipids (*p *=* *0.03) and triacylglycerols (TAG) (*p *=* *0.02), but less hepatic phosphatidylcholine (*p *=* *0.02) compared to those fed the non‐CFAM diets. CFAM did not alter liver phosphatidylethanolamine *N*‐methyltransferase (PEMT) activity and CTP: phosphocholine cytidylyltransferase (CT‐α) protein levels. Rats fed CFAM diets had higher levels of plasma total cholesterol (TC), VLDL + LDL cholesterol, higher ratio of TC to HDL cholesterol, and lower levels of HDL cholesterol compared with rats fed non‐CFAM diets (*p *<* *0.05). Plasma alanine transaminase (ALT) was decreased with CFAM, but plasma insulin, glucose, and TAG did not vary among the four diet groups (*p *<* *0.05). Rats fed canola oil and CFAM had higher plasma levels of aspartate transaminase (AST) and AST/ALT ratio compared with the other three diet groups. These results indicate that CFAM may provoke an accumulation of TAG in the liver related to a decrease in phosphatidylcholine (PC) levels, but the effect of CFAM on PC concentrations may not occur through impairment of the two main PC biosynthesis pathways.

## 1 INTRODUCTION

Hepatic accumulation of fat is an early stage of progression to nonalcoholic fatty liver disease (NAFLD) (Vanni et al., 2010). Lipid accumulation in the liver can result from increased adipose lipolysis, increased hepatic de novo lipogenesis (DNL), impaired synthesis and/or secretion of VLDL cholesterol, triacylglycerol (TAG) esterification dysfunction, or impaired mitochondrial β‐oxidation (Kawano & Cohen, 2013). Typical features of hepatic steatosis and NAFLD may include elevated TAG and fasting plasma glucose, increased or normal alanine transaminase (ALT), and low HDL cholesterol and can lead to insulin resistance (IR), obesity, and type 2 diabetes mellitus (T2DM) (Yki‐Jarvinen, 2014). However, the extent of these metabolic manifestations differs with respect to dietary fat type. For instance, diets high in *trans* fatty acids can increase visceral fat, liver lipid accumulation (Dorfman et al., 2009), and low‐density lipoprotein (LDL) cholesterol and decrease high‐density lipoprotein (HDL) cholesterol (Lichtenstein et al., 1999). In hamsters, it has been found that a combination of low monounsaturated fatty acid (MUFA) and low polyunsaturated fatty acid (PUFA) to saturated fatty acid (SFA) ratio induces weight gain and body fat accumulation, while a high MUFA and high PUFA/SFA ratio prevent white adipose tissue accumulation (Liao et al., 2010).

Frying and the industrial refining of vegetable oils can generate cyclic fatty acid monomers (CFAM) which are 18‐carbon unsaturated fatty acids with internal ring structure formed mainly from linoleic and α‐linolenic acids during heating of edible vegetable oils at temperatures of ≥200°C. CFAM generated from 18:3(*n* − 3) are composed of 16 identified isomers, with a mixture of C5‐ and C6‐membered rings (Dobson et al., 1995). Heated edible oils can contain up to 0.66% CFAM of total fatty acids (Frankel et al., 1984). It has been observed that diets containing CFAM increase either liver weight or hepatic lipid content compared to control diets (Iwaoka & Perkins, 1976), but the underlying mechanisms are not fully elucidated. Martin et al. (2000) already reported that dietary CFAM can increase the activities of liver enzymes involved in peroxisomal β‐oxidation (acyl‐CoA oxidase, (ω‐1)‐laurate hydroxylase, and ω‐laurate hydroxylase). However, *in vivo* studies that addressed the metabolic effects of CFAM in rats did not document a combined effect of CFAM and different types of dietary fat on the levels and types of lipid accumulated in the liver.

Phosphatidylcholine, phosphatidylethanolamine (PE), and the molecular ratio of PC/PE play a critical role in liver health and disease (van der Veen et al., 2017). Hepatic PC content is required both for the assembly and secretion of very low‐density lipoprotein (VLDL) and chylomicrons (Skipski et al., 1967). Therefore, as shown in mice, PC protects against the development of steatosis (Niebergall & Vance, 2012) and a decrease in liver PC has been observed in human cases of hepatic steatosis (Puri et al., 2007). Moreover, a recent review (van der Veen et al., 2017) highlighted the importance of the cellular molar PC/PE ratio in the development of hepatic steatosis. Indeed, relatively small or larger reductions in the PC/PE molar ratio may contribute to the development of NAFLD. In the liver, PC is biosynthesized, mainly, through the CDP‐choline pathway which is regulated by CTP: phosphocholine cytidylyltransferase (CT‐α) (Vance & Vance, 2008), and via three sequential methylations of PE by PE *N*‐methyltransferase (PEMT) (Vance & Ridgway, 1988). Mice lacking hepatic CT‐α or PEMT develop nonalcoholic fatty liver disease (NAFLD), which evolves to nonalcoholic steatohepatitis, when mice are challenged with a high‐fat diet (van der Veen et al., 2017). These observations show how both pathways are critical to liver health. However, to our knowledge, there are no studies assessing how dietary lipids, such as CFAM, affect either of these two pathways.

Due to unresolved issues related to the effects of CFAM on hepatic fat accumulation, the objectives of this study were to assess the effects of CFAM on hepatic TAG, cholesterol, and phospholipids and to investigate whether these effects are modulated by two different dietary fats. Canola and soybean oils were used in this study because of their different fatty acid profiles as well as their different *n* − 6/*n* − 3 ratios (canola oil: 2.2, soybean oil: 7.8), which have been shown to have different impacts on lipid metabolism in rodents (Lee et al., 1989; Liao et al., 2010). This study also focused on the activity of PEMT and protein expression of CT, the two hepatic enzymes involved in phosphatidylcholine synthesis, and other critical components of metabolic lipid processes, after CFAM administration. We hypothesized that dietary CFAM supplementation may increase TAG accumulation in the liver associated with impaired PC biosynthesis.

## 2 MATERIALS AND METHODS

### 2.1 CFAM extraction from heated linseed oil

Cyclic fatty acid monomers were extracted from heated linseed oil as previously described (Sebedio et al., 1987). In brief, linseed oil (Maison Orphée, Quebec City, QC, Canada) was heated in sealed tubes under nitrogen in a HP chromatograph oven (HP 68900 series) at 275°C for 12 hr. After saponification and methylation, CFAM were isolated by a combination of column chromatography and urea fractionation. Confirmation of CFAM isomers was performed by gas chromatography–mass spectrometry (GC‐MS) after picolinyl esters of CFAM were synthesized as previously described (Destaillats & Angers, 2002) and determination of each isomer of the 5‐membered and 6‐membered rings CFAM was by GC (Dobson et al., 1995). GC‐MS analyses for the confirmation of CFAM isomers structures were performed using a gas chromatograph (Hewlett‐Packard, Model 5890, Series II, Palo Alto, CA, USA) coupled with a selective quadrupole mass detector (Agilent, model 5973 N, Palo Alto, CA). Later, CFAM‐methyl esters were converted to free fatty acids by saponification (ethanol 95% and KOH) overnight at room temperature. The CFAM‐free fatty acids were filtered with 2% bentonite, and peroxides were neutralized with NaS_2_O_3_. The extracts were kept at −20°C until incorporation in the diet.

### 2.2 Experimental diets

Table 1 presents the formulation of the four (4) diet groups based on a combination of the type of oil (canola or soybean) and the dose of CFAM (0.0% and 0.5% of total dietary lipids): canola oil (CO), canola and CFAM (CC), soybean oil (SO), and soybean and CFAM (SC). Fatty acid profiles of the diets were determined by gas chromatography (GC) and are also presented in Table 1. The diets were formulated to be isoenergetic, isolipidic and isonitrogenous. Total energy in diets was determined with an adiabatic Parr 6300 calorimeter (Parr Instrument Company, Moline, IL, USA) and was similar among the four diets (CO, 19.5 kJ/g; CC, 19.4 kJ/g; SO, 19.5 kJ/g; SC, 19.5 kJ/g). Dietary protein content was determined by combustion (Dumas method) using a LECO FP‐528 apparatus (LECO Corporation, St. Joseph, MI, USA) and was also found similar between the diets (CO, 19.3% [w/w]; CC, 19.3% [w/w]; SO, 18.9% [w/w]; SC, 19.7% [w/w]). Dietary fat content was measured with an ANKOM^XT10^ Extractor (ANKOM Technology, Macedon, NY, USA) and was similar between the diets (CO, 10.1% [w/w]; CC, 10.3% [w/w]; SO, 10.0% [w/w]; SC, 10.2% [w/w]).

**Table 1:** Formulation (g/kg) and fatty acid composition (weight%) in canola oil (CO), canola + CFAM (CC), soybean oil (SO), and soybean + CFAM (SC) diets

	CO	CC	SO	SC
Casein	180	180	180	180
Sucrose	200	200	200	200
Maize starch	420	420	420	420
Cellulose	50	50	50	50
l‐Cysteine	3	3	3	3
Choline bitartrate	2	2	2	2
Vitamins mix (AIN‐93‐VX)	10	10	10	10
Mineral mix (AIN‐93G‐MX)	35	35	35	35
Lipids including CFAM (+0.02% TBHQ)	100	100	100	100
Fatty acid composition				
Total SFA	4.8	4.8	13.2	13.2
Total MUFA	61.3	61.3	20.5	20.5
Total *n *− 6 PUFA	17.8	17.8	52.0	52.0
Total *n *− 3 PUFA	8.0	8.0	6.7	6.7
*n *− 6/*n *– 3	2.2	2.2	7.8	7.8
CFAM^a^	0	0.5	0	0.5

Notes

CFAM: cyclic fatty acid monomers; MUFA: monounsaturated fatty acids; PUFA: polyunsaturated fatty acids; SFA: saturated fatty acids; TBHQ: tert‐butylhydroquinone.

a The CFAM fraction of the diet contained 56.8% of 5‐membered ring isomers and 43.2% of 6‐membered ring isomers of the total CFAM (see Table 2 for a detailed description of the 16 CFAM isomers).

### 2.3 Animals and experimental design

Thirty‐six (36) male Wistar rats initially weighing *ca*. 150 g were housed individually in solid‐bottom cages with bedding to absorb water and urine. The animal room was maintained at constant temperature (22 ± 2°C) and humidity (45%–55%), and the rats were kept at an inverted light–dark cycle with the light being from 07.00 a.m. to 07.00 p.m. Animals were acclimated to their new environment for 7 days and fed a rat chow (Purina Canada, Mississauga, ON) and had access to tap water *ad libitum*. After acclimation, rats were housed in individual cages, divided into four purified diet groups (Table 2) according to a 2 × 2 factorial experimental design.

**Table 2:** Isomer composition (mol%) of the CFAM fraction in canola oil + CFAM (CC) and soybean oil + CFAM (SC) dietsa,b

Peak	Ring			Double bonds		% Of total CFAM
	Size^a^	Position^a^	Config.^b^	Position^a^	Config.^b^	
a	5	10–14	*trans*	12, 15	*E*	10.7
b	5	11–15	*trans*	9, 12	*E*	5.8
c	5	11–15	*trans*	9, 12	*Z*	1.1
d	5	10–14	*trans*	12, 15	*Z*	6.4
d′	5	10–14	*cis*	12, 15	*E*	7.4
e	5	11–15	*cis*	9, 12	*E*	0.1
f	5	10–14	*cis*	12, 15	*Z*	15.3
g	5	11–15	*cis*	9, 12	*Z*	10.0
h	6	10–15	*cis*	8, 12	*E*	6.4
i	6	10–15	*trans*	8, 12	*E*	12.6
j	6	10–15	*trans*	8, 12	*Z*	0.8
k	6	10–15	*trans*	12, 16	*E*	8.7
l	6	10–15	*cis*	8, 12	*Z*	13.2
m	6	10–15	*cis*	12, 16	*E*	0.7
n	6	10–15	*trans*	12, 16	*Z*	0.2
o	6	10–15	*cis*	12, 16	*Z*	0.6

^a^Ring size and position, and position of acyclic double bond were determined by GC‐MS of picolinyl derivatives. ^b^Ring and acyclic double bond configurations were determined based on Dobson et al. (1995). Configuration of endocyclic double bond is *Z*.

After acclimation, animals were put on a 7‐day adaptation period during which they were given a gradual percentage increase in the purified diet to the chow diet (25–75 and 50–50 for 2 days each, and 75–25 for 3 days). This was followed by a 28‐day 100% purified diet experiment. Food consumption and body weight were recorded daily, while water was given *ad libitum*. Food efficiency was calculated according to the following equation:

$$\text{Food efficiency}=\frac{\text{grams of daily weight gain}}{\text{grams of daily food intake}}$$

Three weeks through the experiment, animals were placed in metabolic cages, and their urine and feces were collected daily. At the end of the 28‐day period, all animals were sacrificed in a 12‐hr fasting state by cardiac puncture under isoflurane USP (Pharmaceutical Partners Canada, Richmond Hill, ON). Livers were excised, weighed, and stored directly in liquid nitrogen and thereafter at −80°C until analysis. After emptying the gastrointestinal tract, animal carcasses were autoclaved, ground, lyophilized, and ground for body composition analyses. Treatment procedures and animal care were approved by the Laval University Animal Care Committee in accordance with the Canadian Council on Animal Care guidelines (file #2015‐004).

### 2.4 Body composition

For each rat, body composition and total energy was determined according to a previously described method by Jacques et al, (2010). Rat liver protein was determined by LECO FP‐528 (St. Joseph, MI, USA) according to the Dumas method.

### 2.5 Liver lipid extraction

Liver lipid extraction was performed according to Folch and collaborators (Folch et al., 1957). In brief, *ca*. 0.5 g of liver were weighed and ground with chloroform/methanol (2:1, by vol) using a grinder (Fisher stirrer Dyna‐Mix 43, Fisher Scientific, USA). After addition of an aqueous solution of NaCl 0.78% and shaking, the mixture was left overnight for complete separation of the two phases. The lower phase containing total lipids was extracted, and lipids were determined gravimetrically after evaporation of solvent under nitrogen. Liver lipids were later stored at −20°C for lipid classes and fatty acid analysis.

### 2.6 Liver total cholesterol, triacylglycerols, phosphatidylethanolamine, and phosphatidylcholine

Total liver lipids were separated into classes by HPLC‐ELSD according to a method previously described (Morin et al., 2004). In brief, total lipids were dissolved in a chloroform/methanol mixture (2:1 by vol.) to a final concentration of 2 mg/ml. Ten microlitre were injected into a Waters HPLC Autosampler Model 717 plus (Waters, Milford, MA, USA) connected to a Sedex 75 ELSD detector (Sedere, Alfortville, France). The column used was a Zorbax SIL 4.6 mm ID × 150 mm (5 μm) (Agilent Technologies, Mississauga, ON, Canada). Chloroform and methanol were used as solvents.

### 2.7 Liver fatty acid composition

Prior to gas chromatography (GC) analysis, fatty acid methyl esters were prepared by base‐catalyzed transesterification. To 10 mg of total lipids were added 1 ml hexane and 500 μl CH_3_ONa 0.5 N (Sigma‐Aldrich, St. Louis, MO, USA). After vortexing, the mixture was put in a water bath for 15 min at 40–50°C. 4 ml hexane and 5 ml H_2_O saturated in NaCl were added and the vial was vortexed and left still at room temperature for the separation of the two phases. The upper phase was collected, filtered, and dried in a Pasteur pipette containing fat‐free wool and *ca*. 1 cm of MgSO_4_. FAME were quantified by GC with a Shimadzu GC‐2010 apparatus (Santa Clara, CA, USA) equipped with a flame ionization detector (FID) at 250°C connected to a computer with a Shimadzu GC‐Solution software. The injector temperature was kept at 250°C. Hydrogen was used as the carrier gas under a constant flow (1.29 ml/min). Sample volumes (1.0 μl) were injected with a split ratio of 50 on a 60 m × 0.25 mm i.d. × 0.25 μm (film thickness) BPX‐70 column (SGE, Melbourne, Australia). Temperature programming mode consisted of 60°C isothermal for 1 min, increased to 190°C at 10°C/min, isothermal for 15 min, increased to 240°C at 5°C/min, and held for 6 min at this temperature for a total run of 45 min. Individual FAME were identified by comparison with known standards retention time (GC Shimadzu FAME Method RC‐32 cm/s). Liver CFAM content was determined in the same conditions.

### 2.8 Liver glycogen

Liver glycogen was determined by a coupled enzyme assay, and the absorbance was measured by colorimetry at 570 nm wavelength using a commercial assay kit (Sigma‐Aldrich). In brief, *ca*. 10 mg liver were homogenized in 100 μl H_2_O on ice, boiled for 5 min, and centrifuged at 13,000 *g* for 5 min. Glycogen was determined in 30 μl of the supernatant.

### 2.9 Liver PEMT activity and CT‐α protein analysis

Liver phosphatidylethanolamine *N*‐methyltransferase (PEMT) activity was determined from 5 μg of liver homogenate protein according to Jacobs and coworkers (Jacobs et al., 2004). CTP: phosphocholine cytidylyltransferase (CT‐α) protein expression was determined by Western blotting as previously described in a previous study (da Silva et al., 2015).

### 2.10 Liver histology and assessment of fat accumulation

Working on dry ice, liver samples were taken from whole liver after immersion in liquid nitrogen. Liver samples were later weighed and preserved in 10% phosphate‐buffered formalin, pH 7.0. before staining with hematoxylin and eosin (H&E) by the pathology service of the Quebec Heart and Lung Institute. Images were captured at ×20 magnification using the Zeiss Axio Observer Z1 microscope (Carl Zeiss, Oberkochen, Germany).

### 2.11 Plasma analyses

#### 2.11.1 Fasted triacylglycerols, total cholesterol, VLDL + LDL cholesterol, and HDL cholesterol

Triacylglycerols were measured by a colorimetric assay kit (Cayman Chemical, Ann Arbor, MI, USA) using the hydrolysis of TAG to glycerol and free fatty acids, and the absorbance of glycerol was measured at 540 nm. Total cholesterol was measured by a fluorometric method using a commercial kit (Cayman Chemical). VLDL + LDL cholesterol and HDL cholesterol were measured with a colorimetric assay kit (Abcam, Cambridge, MA, USA).

#### 2.11.2 Fasted glucose and insulin

Glucose in the plasma was measured with a colorimetric assay kit (Cayman Chemical) using the glucose oxidase–peroxide reaction, and glucose concentration was determined by measuring the absorption at 514 nm. Insulin was measured by ELISA using a commercial kit (EMD Millipore, Saint Charles, MO, USA).

#### 2.11.3 Fasted ALT and AST

Alanine transaminase activity was measured by a colorimetric assay kit (Cayman Chemical) by monitoring the oxidation of NADH to NAD^+^ at the absorbance of 340 nm. Aspartate transaminase (AST) activity was measured by a colorimetric assay kit (Sigma‐Aldrich) according to the manufacturer's specifications, and absorbance was measured at 450 nm.

### 2.12 Statistical analysis

All data are expressed as mean ± *SEM*. ANOVA for 2 × 2 factorial experimental design was performed with SAS software (SAS Institute Inc., Cary, NC, USA) to determine the CFAM and oil effects, as well as interactions among CFAM and dietary oils. When significant CFAM–oil interactions were detected, a Multiple Comparison Test, that is, Tukey Studentized Range test (HSD), was performed to identify differences among diet groups. Pearson correlation coefficients were calculated between variables, using the CORR procedure (SAS). The tests were considered significant at *p *≤* *0.05.

## 3 RESULTS

### 3.1 CFAM production

**Figure 1:**
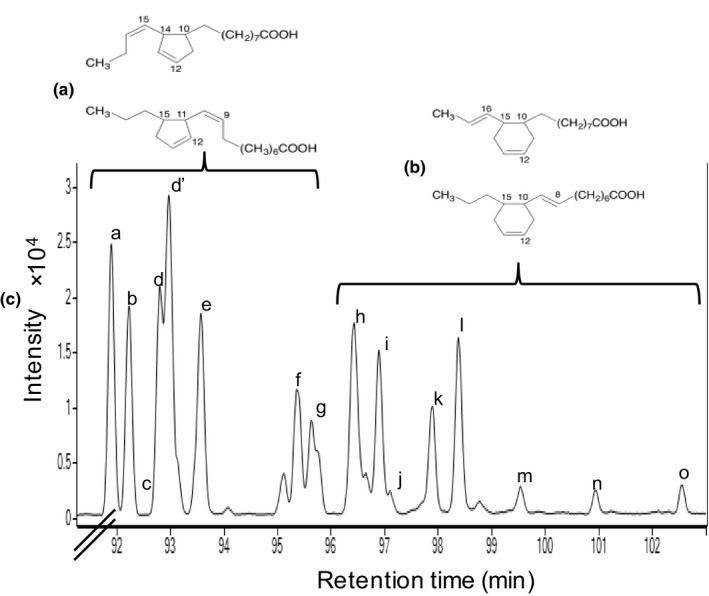
Representative structures of cyclopentenyl (a) and cyclohexenyl (b) CFAM isomers from α‐linolenic acid, formed during heat treatment. The chromatogram (c) shows the peaks of the 16 cyclopentyl (peaks a–g) and cyclohexenyl (peaks h–o) CFAM isomers. CFAM: cyclic fatty acid monomers

The CFAM fraction was composed of 16 identified isomers. The cyclopentenyl isomers accounted for 56.8% of the fraction, while the cyclohexenyl isomers accounted for the remaining 43.2% (Table 2). The CFAM fraction used in this study was therefore a mixture of both the 5‐membered and 6‐membered rings isomers (Figure 1).

### 3.2 Effects of diets on food consumption, food efficiency, body weight gain, and composition

Data for food consumption, weight gain, food efficiency, and body composition as well as liver protein, glycogen, and cholesterol are shown in Table 3. At the end of the 28‐day experimental period, no differences were found between the 4 groups for food consumption and weight gain. Although CFAM did not alter food efficiency, rats fed soybean oil had a higher food efficiency compared with the canola oil group (*p* = 0.02). Regarding body composition, rats fed the four diets had similar lean and fat mass. However, rats fed soybean oil had significantly lower total body energy content than those fed canola oil (*p *=* *0.03). No difference was observed for liver protein, glycogen, and cholesterol.

**Table 3:** Food consumption, growth, liver and fecal lipids, glucose, insulin, and body composition of rats fed canola oil (CO), canola + CFAM (CC), soybean oil (SO), and soybean + CFAM (SC)

	Diets				*p* Values	
	CO	CC	SO	SC	OIL	CFAM
Initial weight (g)	274 ± 4	280 ± 4	275 ± 4	277 ± 3	NS	NS
Final weight (g)	469 ± 10	479 ± 8	477 ± 7	489 ± 11	NS	NS
Food consumption (g/day)	26.7 ± 0.5	27.1 ± 0.5	26.6 ± 0.5	27.1 ± 0.6	NS	NS
Weight gain (g/4 weeks)	194 ± 8	199 ± 8	202 ± 4	212 ± 8	NS	NS
Food efficiency (g/g)	0.26 ± 0.0	0.26 ± 0.0	0.27 ± 0.0	0.28 ± 0.0	*	NS
Liver measurements						
Liver (g)	16.4 ± 0.4	17.8 ± 0.6	16.1 ± 0.5	17.5 ± 0.9	NS	*
Relative liver weight (g/100 g)	3.5 ± 0.0	3.7 ± 0.0	3.4 ± 0.0	3.6 ± 0.0	NS	NS
Liver protein (g)	11.6 ± 0.2	11.6 ± 0.4	11.5 ± 0.3	12.3 ± 0.4	NS	NS
Liver glycogen (mg/g protein)	230 ± 16	236 ± 18	259 ± 26	180 ± 21	NS	NS
Liver total cholesterol (mg/g protein)	9.9 ± 0.6	11.4 ± 0.8	9.2 ± 0.4	8.9 ± 1	NS	NS
Plasma measurements (in fasted state)						
Glucose (mg/dl)	87 ± 5	83 ± 6	102 ± 8	102 ± 8	NS	NS
Insulin (ng/ml)	1.2 ± 0.1	1.6 ± 0.4	1.5 ± 0.4	1.4 ± 0.3	NS	NS
Body carcass composition						
Total energy (kJ/g)	28.2 ± 0.3	28.4 ± 0.1	27.8 ± 0.3	27.4 ± 0.3	*	NS
Body fat mass (mg/g)	440 ± 20	452 ± 7	414 ± 16	421 ± 17	NS	NS
Lean body mass (mg/g)	463 ± 23	450 ± 10	489 ± 15	460 ± 18	NS	NS

NS: not significant.

Data are mean ± *SEM* (ANOVA for 2 × 2 factorial experimental design, *p *<* *0.05, *n* = 9). *p < 0.05.

### 3.3 Effects of CFAM and non‐CFAM diets on liver lipid composition and function

**Figure 2:**
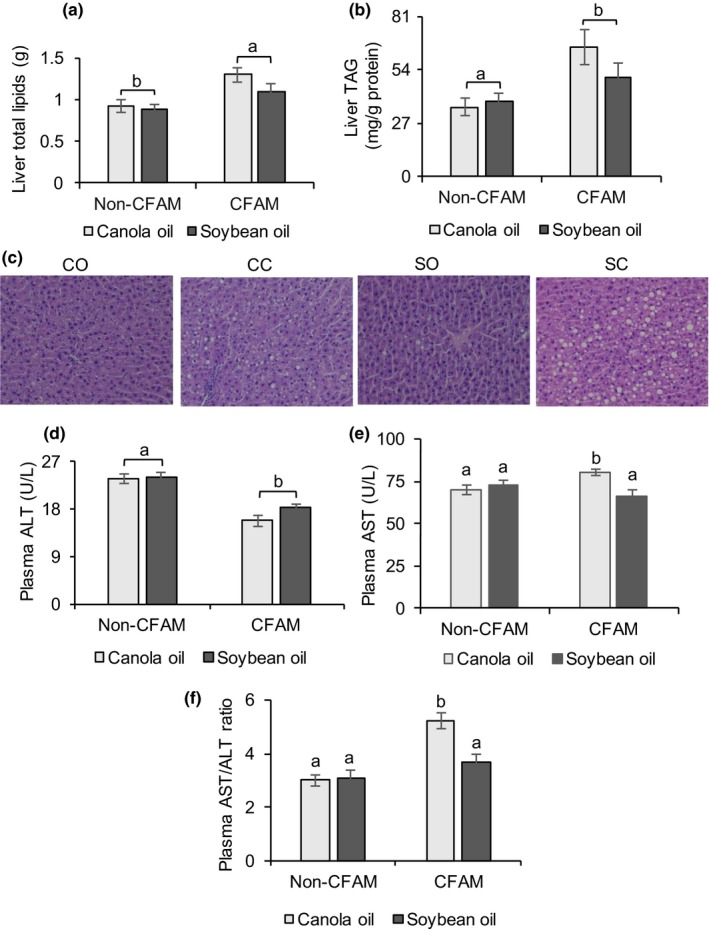
Liver total lipids (a), TAG (b), and histological samples fixed in buffered formalin and stained with hematoxylin and eosin (c) and plasma ALT (d), AST (e), and AST/ALT ratio (f) of rats fed either the non‐CFAM or CFAM diets. All data are means ± *SEM*. Bars with differing small letters are significantly different (ANOVA for 2 × 2 factorial experimental design, *p *<* *0.05, *n* = 9). ALT: alanine transaminase; AST: aspartate transaminase; CFAM: cyclic fatty acid monomers; TAG: triacylglycerols

Cyclic fatty acid monomer diets did not affect relative liver weight, that is, the ratio of liver weight to final body weight (Table 3). Liver total lipids (*p *=* *0.03, Figure 2a) and TAG (*p *=* *0.02, Figure 2b) were higher in rats fed CFAM diets compared to those fed the non‐CFAM diets. Fats deposits from histological samples stained with H&E were visible in the livers of rats fed the CFAM diets (Figure 2c). Alanine transaminase (ALT) was reduced in the plasma of rats fed CFAM compared to the non‐CFAM diet groups (*p *<* *0.0001, Figure 2d). There were significant interactions between oil and CFAM on plasma alanine transaminase (AST) and AST/ALT ratio (*p *<* *0.05). Rats fed canola oil and CFAM had higher plasma AST levels (*p *=* *0.04, Figure 2e) and AST/ALT ratio (*p *=* *0.02, Figure 2f) compared with the other three diet groups.

### 3.4 Effects of diets on liver lipids and phosphatidylcholine biosynthesis enzymes

**Figure 3:**
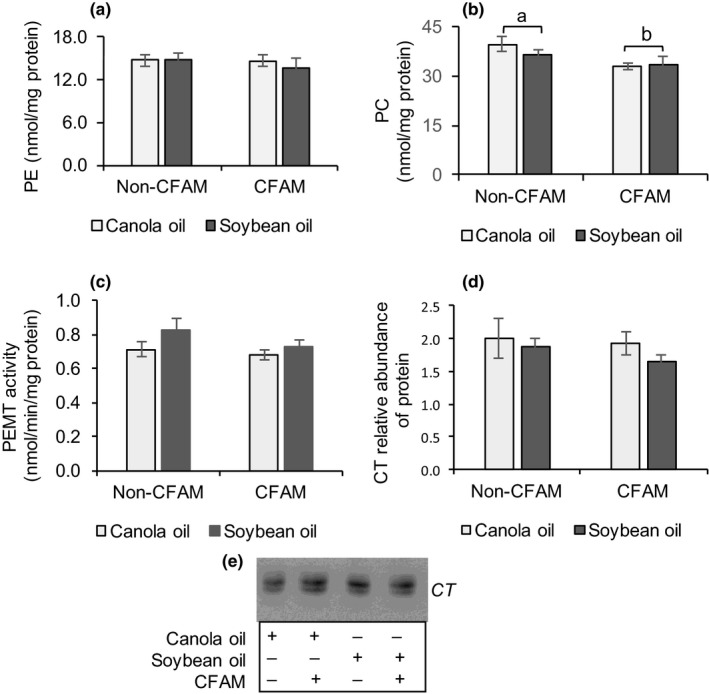
Liver PE (a), PC (b), PEMT activity (c), CT protein expression (d), and representative immunoblots for the expression of CT (e) of the livers of rats fed canola oil or soybean oil with or without 0.5% CFAM diets. All data are means ± *SEM*. (ANOVA for 2 × 2 factorial experimental design, *p *<* *0.05, *n* = 9). Bars with differing letters are significantly different. CT: CTP: phosphocholine cytidylyltransferase; PC: phosphatidylcholine; PE: phosphatidylethanolamine; PEMT: phosphatidylethanolamine *N*‐methyltransferase

No differences were observed for liver phosphatidylethanolamine (PE, Figure 3a), PEMT activity (Figure 3c), and CT protein (Figure 3d). The CFAM diets induced lower phosphatidylcholine levels (*p *=* *0.02, Figure 3b) in the liver compared with the non‐CFAM diets. There was no significant difference in the PC/PE molar ratio in rats fed the CFAM diets compared to those fed the non‐CFAM diets (2.4 ± 0.1 for the CFAM diet groups, and 2.6 ± 0.1 for the non‐CFAM diet groups).

### 3.5 Effects of CFAM and non‐CFAM diets on liver fatty acid composition

Liver fatty acid composition is presented in Table 4. Rats fed canola oil had more myristic acid (14:0), oleic acid (18:1*n *− 9), vaccenic acid (18:1*n *− 7), eicosapentaenoic acid (EPA, 20:5*n *− 3), and total monounsaturated fatty acids (MUFA) than those fed soybean oil. These had more linoleic acid (18:2*n *− 6), α‐linolenic acid (18:3*n *− 3), gondoic acid (20:1*n *− 9), 20:2*n *− 6, dihomo‐γ‐linolenic acid (20:3*n *− 6), lignoceric acid (24:0), arachidonic acid (20:4*n *− 6), total *n *− 6 PUFA, and total PUFA than the former. The CFAM diets increased palmitelaidic acid (PEA, 16:1 9t) levels compared with the non‐CFAM diets. There was also an oil effect for this same fatty acid, with canola oil inducing more PEA than soybean oil. When the two CFAM diets were compared, rats fed the CC diet had roughly double the amount of CFAM in their livers compared with those fed the SC diet (*p *=* *0.0009).

**Table 4:** Fatty acid composition (weight %) in total liver lipids of rats fed diets containing canola oil (CO), canola oil and CFAM (CC), soybean oil (SO), and soybean oil and CFAM (SC)

Fatty acid	Diets				*p* Values	
	CO	CC	SO	SC	OIL	CFAM
14:0	0.5 ± 0.02	0.7 ± 0.04	0.5 ± 0.06	0.5 ± 0.06	*	NS
16:0	23.5 ± 0.5	25.8 ± 1.4	22.4 ± 0.9	22.6 ± 1.3	NS	NS
16:1 9t	0.6 ± 0.04	0.8 ± 0.04	0.3 ± 0.03	0.4 ± 0.04	**	*
16:1 9c	2.2 ± 0.1	3.2 ± 0.4	2.0 ± 0.4	2.2 ± 0.5	NS	NS
18:0	12.3 ± 0.6	9.2 ± 0.7	11.2 ± 0.8	11.7 ± 0.9	NS	NS
18:1 (*n *− 9)	26.5 ± 1.3	31.8 ± 1.4	13.9 ± 1.4	13.1 ± 1.4	**	NS
18:1 (*n *− 7)	3.0 ± 0.1	3.1 ± 0.2	2.6 ± 0.1	2.7 ± 0.2	*	NS
18:2 (*n *− 6)	9.7 ± 0.3	8.9 ± 0.8	23.4 ± 1.5	22.9 ± 2.2	**	NS
18:3 (*n *− 6)	0.3 ± 0.07	0.3 ± 0.03	0.4 ± 0.07	0.5 ± 0.1	NS	NS
18:3 (*n *− 3)	0.7 ± 0.1	0.8 ± 0.1	1.5 ± 0.2	1.4 ± 0.3	**	NS
20:1 (*n *− 9)	0.2 ± 0.01	0.2 ± 0.03	0.2 ± 0.02	0.2 ± 0.02	*	NS
20:2 (*n *− 6)	0.08 ± 0.0	0.09 ± 0.0	0.3 ± 0.02	0.25 ± 0.02	**	NS
20:3 (*n *− 6)	0.4 ± 0.04	0.4 ± 0.04	0.3 ± 0.02	0.3 ± 0.03	*	NS
20:4 (*n *− 6)	13.6 ± 0.8	9.6 ± 0.9	14.6 ± 1.2	15.0 ± 1.2	*	NS
24:0	0.13 ± 0.01	0.1 ± 0.01	0.3 ± 0.05	0.3 ± 0.02	**	NS
20:5 (*n *− 3)	0.5 ± 0.04	0.6 ± 0.07	0.4 ± 0.04	0.5 ± 0.07	*	NS
22:5 (*n *− 6)	0.7 ± 0.03	0.6 ± 0.05	0.7 ± 0.06	0.7 ± 0.06	NS	NS
22:6 (*n *− 3)	5.3 ± 0.3	3.8 ± 0.3	5.1 ± 0.5	4.9 ± 0.4	NS	NS
CFAM[Link]	0.0	0.12 ± 0.02^a^	0.0	0.05 ± 0.01^b^		
∑SFA[Link]	36.5 ± 0.7	35.8 ± 1.2	34.4 ± 1.0	35.1 ± 1.3	NS	NS
∑MUFA[Link]	32.5 ± 1.5	39.2 ± 1.7	18.9 ± 1.8	18.5 ± 2.0	**	NS
∑PUFA *n *− 6[Link]	23.7 ± 1	19.3 ± 1.5	39.0 ± 1.7	38.9 ± 2.4	**	NS
∑PUFA *n *− 3[Link]	7.3 ± 0.4	5.9 ± 0.4	7.7 ± 0.5	7.4 ± 0.5	NS	NS
∑PUFA[Link]	30.9 ± 1.2	25.1 ± 1.9	46.7 ± 2.1	46.4 ± 2.9	**	NS

NS: not significant.

^1^CFAM: cyclic fatty acid monomers; different superscript letter indicates statistical significant difference (*p* = 0.0009). ^2^Sum of the saturated fatty acids. ^3^Sum of the monounsaturated fatty acids. ^4^Sum of the polyunsaturated fatty acids (PUFA) *n* – 6. ^5^Sum of the PUFA *n *– 3. ^6^Sum of the total PUFA.

Data are mean ± *SEM* (ANOVA for 2 × 2 factorial experimental design, *p *<* *0.05, *n* = 9). *p < 0.05, **p < 0.01.

### 3.6 Effects of CFAM and non‐CFAM diets on plasma lipids

**Figure 4:**
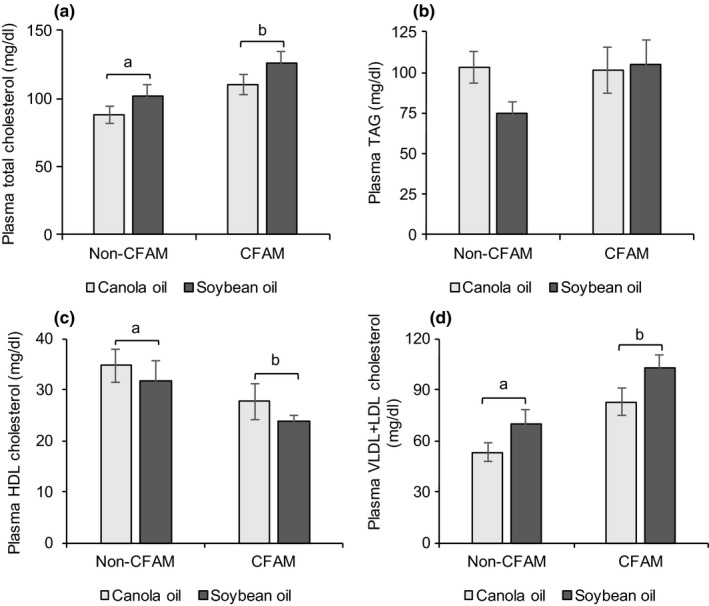
Plasma total cholesterol (a), TAG (b), HDL cholesterol (c), and VLDL + LDL cholesterol (d) of rats fed canola oil or soybean oil with or without 0.5% CFAM diets. All data are means ± *SEM*. (ANOVA for 2 × 2 factorial experimental design, *p *<* *0.05, *n = *9). Bars with differing letters are significantly different. HDL: high‐density lipoprotein; VLDL + LDL: very low‐density lipoprotein plus low‐density lipoprotein; TAG: triacylglycerols

Plasma total cholesterol (Figure 4a), triacylglycerols (Figure 4b), HDL cholesterol (Figure 4c), and VLDL + LDL cholesterol (Figure 4d) are presented in Figure 4. Plasma TAG levels did not vary among the four diet groups. Rats fed the CFAM diets had more plasma total cholesterol (*p *=* *0.03) and VLDL + LDL cholesterol (*p *=* *0.003) than those fed the non‐CFAM diets. In the latter case, there was also an oil effect, whereby rats fed soybean oil had more VLDL + LDL cholesterol than those fed canola oil (*p *=* *0.04). Rats fed the CFAM diets had less HDL cholesterol and higher total cholesterol to HDL cholesterol ratio than those the non‐CFAM diets (*p *=* *0.01 and *p *=* *0.0009, respectively).

### 3.7 Effects of diets on plasma biochemical parameters

No differences were observed for plasma fasting glucose and insulin (Table 3). However, there was a strong tendency (*p *=* *0.054) for the rats fed the soybean diets with or without CFAM to induce higher fasting glucose compared with those fed the canola diets with or without CFAM.

## 4 DISCUSSION

In the past decades, the metabolic effects of dietary oils and certain types of fatty acids have been fully described, showing therefore that those oils and fatty acids can be associated with lipid improvements or disturbances. Although there exists some evidence that dietary CFAM have a negative impact on the development of fatty liver (Lamboni et al., 1998), evidence on the underlying mechanisms leading up to fat accumulation in the liver is still limited. To the best of our knowledge, this is the first study to assess the impact of CFAM combined with two different dietary oils on markers of hepatic steatosis in relative low‐fat diet fed rats. This study compared hepatic lipid and fatty acid response as well as liver function and phosphatidylethanolamine *N*‐methyltransferase (PEMT) activity and CTP: phosphocholine cytidylyltransferase (CT‐α) protein expression and plasma lipid response across four 10% (w/w) fat diets: two diets based on soybean oil with or without 0.5% (w/w) CFAM, and two based on canola oil with or without 0.5% (w/w) CFAM. The soybean oil diets consisted primarily of linoleic acid, whereas the canola oil diets consisted primarily of oleic acid. Thus, the untoward metabolic responses observed with the CFAM diets, which included the presence of CFAM in the liver, increased liver TAG, decreased liver PC, increased plasma VLDL + LDL cholesterol, and decreased HDL cholesterol, were observed in both the soybean and canola oil‐based diets and were likely due to the presence of CFAM in the diets. There were no changes in PEMT activity and CT‐α protein.

Most of the fatty acid modifications in the liver were due to the oil consumption. As expected, rats fed the canola oil diets had more total MUFA and oleic acid in the liver than those fed the soybean oil diets, which in turn had more linoleic, arachidonic, α‐linolenic acids. These differences can be explained both by the fatty acid composition and the difference in *n *− 6/*n *− 3 ratio of the diets (Takeuchi et al., 2001). Besides, differences were found in the accumulation of CFAM in the liver of rats, with those fed canola oil and CFAM accumulating roughly double the amount of those fed soybean and CFAM. Joffre et al. (2004) reported that CFAM are β‐oxidized at a lower rate compared to linoleic or α‐linolenic acids. Other studies have observed that feeding Wistar rats diets rich in oleic acid (74.8%–79.2% of total dietary fatty acids) for 4 weeks or more resulted in high hepatic levels of lipids and increased de novo lipogenesis (Portillo et al., 2001; Ruiz‐Gutierrez et al., 1999; Takeuchi et al., 2001). Taken together, these observations suggest that high hepatic CFAM levels in rats fed a high‐oleic acid diet (canola oil) containing CFAM could likely be a result of combined effects of both the lower β‐oxidation rate of CFAM and the lipogenic effect of a high‐oleic acid diet.

**Figure 5:**
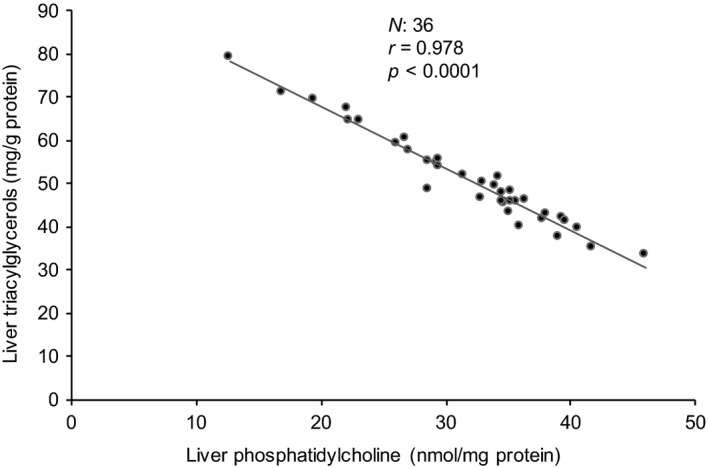
Pearson correlation between liver triacylglycerols and phosphatidylcholine of rats fed canola oil or soybean oil with or without 0.5% cyclic fatty acid monomers diets. All data are means ± *SEM*

Increased liver lipid content observed with both CFAM diets in this study is consistent with the literature (Iwaoka & Perkins, 1976; Lamboni et al., 1998), but we found no significant effect of CFAM on plasma glucose and insulin. These latter results suggest that lipid accumulation in the liver might not resulted from disturbances in carbohydrate homeostasis (Adeli et al., 2001), due likely to the normal physiological state of rats used in this study. Increased hepatic lipid content is rarely associated with increased cholesterol or phospholipid content, but more often with increased liver TAG (Puri et al., 2007). In this study, only an increase in TAG contributed to the increase in total hepatic fat in the CFAM groups. We also noted a strong negative correlation (Figure 5) between hepatic TAG and PC (*r* = −0.978, *p *<* *0.0001), suggesting that hepatic TAG accumulation with CFAM feeding is related to PC metabolism. Indeed, a decrease in PC also occurred along with increased CFAM consumption. In this respect, an inverse relationship was found between liver CFAM and PC (*r* = −0.35, *p *=* *0.036) and PE (*r* = −0.36, *p *=* *0.03) concentrations, suggesting a deleterious impact of CFAM on hepatic phospholipid levels. Because the increases of both hepatic CFAM and TAG were closely associated with hepatic PC, it was plausible that the levels of increased TAG by CFAM in our experimental conditions were partly dictated by decreased PC concentrations. Decreased PC levels can trigger the relocation of SREBP‐activating proteases resulting in the activation of SREBP‐1 and transition to the nucleus, and the upregulation of lipogenesis (Walker et al., 2011). However, in the present study, no significant effect of CFAM was noted on hepatic PEMT activity and CT‐α protein, suggesting that the decrease in liver PC levels observed in the present study was independent of PC biosynthesis pathways. Moreover, the PC/PE ratio remained stable in the present study. One of the many roles of the liver is to maintain the normal membrane PC/PE molar ratio by stimulating PC secretion *via* VLDL and HDL and/or PC degradation by phospholipases, leading to hepatic diacylglycerol and TAG production and accumulation (Jacobs et al., 2013). It is therefore conceivable that dietary CFAM, which elevated hepatic TAG and decreased hepatic PC, may also have increased VLDL secretion and/or PC degradation. The foregoing discussion indicates that the accumulation of TAG in the liver of rats fed CFAM diets is related to a decrease in PC levels, but the exact mechanisms linking the two events need further investigation.

Although it was tempting to speculate that the increase in plasma total cholesterol (TC) and VLDL + LDL cholesterol with CFAM might be due to an increased secretion of VLDL, the absence of CFAM effect on plasma TAG suggest that additional processes contributed to the elevated cholesterolemia. Like *trans* fatty acids (TFA) from industrial sources, CFAM are products of vegetable oils, which had been submitted to a heat treatment at high temperatures (above 220°C), that can affect cholesterol homeostasis. It is interesting that feeding humans with TFA from heated vegetable oils resulted in increased blood LDL cholesterol, TC‐to‐HDL cholesterol ratio, and TAG, and decreased HDL cholesterol (Mensink & Katan, 1990; Zock & Katan, 1992). It is noteworthy that of the 16 CFAM isomers identified in our fraction, 8 isomers were of *trans* configuration. Further studies are thus needed to test whether these isomers can alter cholesterol and lipoprotein metabolism similar to TFA. In the case of TFA, the decrease in plasma HDL cholesterol and increase in LDL cholesterol were attributed, respectively, to an increased lipoprotein apoA‐1 catabolism and a decreased LDL apoB‐100 catabolism (Mozaffarian et al., 2006) and an increase in CETP activity (van Tol et al., 1995). More work is required to state whether either of these pathways contributed to the increase in VLDL + LDL cholesterol and decrease in HDL cholesterol noted with CFAM consumption.

Alanine transaminase and aspartate transaminase are most commonly used serum markers to assess liver function or injury (Botros & Sikaris, 2013). Beside the presence of the metabolic syndrome and type 2 diabetes and fasting serum insulin, raised AST levels and AST/ALT ratio are often used as independent predictors of liver fat and NAFLD (Kotronen et al., 2009). In the present study, we found a positive correlation between plasma AST levels and liver TAG (*r* = 0.33, *p *=* *0.05), confirming that increasing AST levels are related to the development of liver steatosis. Besides, AST and AST/ALT ratio were significantly higher with the canola oil and CFAM diet as compared with the other diets, suggesting that this diet is more prone to liver steatosis. On the other hand, the lower levels of ALT found in rats fed CC or soybean oil and CFAM diets suggest that steatohepatitis or injury was not present in either CFAM diet fed animals. It is interesting that Martin et al. (2000) found increased CYP2E1 activity in rats fed CFAM, that has been associated with elevated serum AST/ALT ratio (Lieber, 2004). These observations suggest that increased AST/ALT ratio in rats fed the canola oil and CFAM diet could be associated with increased activity of CYP2E1. However, the absence of such effects in rats fed soybean oil and CFAM underscores the differential effects of CFAM depending on dietary lipids.

The potential limitations of this study are the physiological nature of the diets and the measurement of CT‐α expression without its activity. Studying the CT‐α expression without the activity may be less likely to detect changes in this PC biosynthesis pathway. Although several findings reached statistical significance, it is likely that changes in the lipid composition of the liver and plasma lipid response as well as liver enzymes in our low‐fat fed rats may be less accentuated with respect to obese high‐fat‐fed rats.

This study has shown that a 4‐week consumption of purified cyclic fatty acids monomers from heated vegetable oils in a relative low‐fat diet negatively affects the cardiometabolic health in rats by increasing liver TAG, plasma total, and VLDL + LDL cholesterol and by decreasing PC levels and plasma HDL cholesterol. Overall, these findings indicate that CFAM may induce steatosis and dyslipidemia by so far unknown mechanisms. A better understanding of the mechanisms linking higher levels of liver TAG and lower PC warrants further investigation. On the basis of the reducing effects of CFAM on liver PC without modifying the molar PC/PE ratio, the possibility that CFAM would also increase the secretion of VLDL and/or PC degradation merits further investigation. Moreover, the analysis of inflammatory and oxidative stress markers could also shed light on the effects of CFAM in rats.

## CONFLICT OF INTEREST

The authors declare that they do not have any conflict of interest.

## ETHICAL REVIEW

This study was approved by the Laval University Animal Care Committee in accordance with the Canadian Council on Animal Care guidelines.
